# Rheotaxis in the Ediacaran epibenthic organism *Parvancorina* from South Australia

**DOI:** 10.1038/srep45539

**Published:** 2017-03-30

**Authors:** John R. Paterson, James G. Gehling, Mary L. Droser, Russell D. C. Bicknell

**Affiliations:** 1 Palaeoscience Research Centre, School of Environmental and Rural Science, University of New England, Armidale, NSW 2351, Australia; 2 South Australian Museum, North Terrace, Adelaide, SA 5000, Australia; 3 Department of Earth Sciences, University of California, Riverside, California 92521, USA

## Abstract

Diverse interpretations of Ediacaran organisms arise not only from their enigmatic body plans, but also from confusion surrounding the sedimentary environments they inhabited and the processes responsible for their preservation. Excavation of Ediacaran bedding surfaces of the Rawnsley Quartzite in South Australia has provided the opportunity to study the community structure of the Ediacara biota, as well as the autecology of individual organisms. Analysis of two bedding surfaces preserving large numbers of *Parvancorina* illustrates that individuals display a preferred, unidirectional orientation aligned with current, as indicated by the identified current proxies: tool marks, overfolded edges of *Dickinsonia*, felled fronds and drag structures generated by uprooted frond holdfasts. Taphonomic and morphological evidence suggests that the preferred orientations of *Parvancorina* individuals are not the result of passive current alignment, but represent a rheotactic response at some stage during their life cycle. These results illustrate a previously unrecognized life mode for an Ediacaran organism and arguably the oldest known example of rheotaxis in the fossil record. The morphology and previously suggested phylogenetic affinities of *Parvancorina* are also re-evaluated. Apart from possessing a bilaterally symmetrical body, there are no unequivocal morphological characters to support placement of *Parvancorina* within the Euarthropoda or even the Bilateria.

Body fossil evidence that shows the ability of Ediacaran organisms to physically respond to external stimuli or move within their environment is generally rare^1^. The only broadly accepted evidence of motility and associated behaviours in the Ediacara biota is represented by trace fossils from Canada, China, Namibia, Russia, and South Australia^2^,^3^,^4^,^5^,^6^. Previous attempts to compare Ediacara disc-shaped fossils (e.g., *Aspidella*) with free-swimming medusoids have been discounted in light of evidence that these were either attachment discs for frond-like organisms^7^ or a motile, benthic animal of cnidarian grade^8^. Serial imprints left by flat organisms such as *Dickinsonia* and *Yorgia* have been previously interpreted as multiple decayed organisms or touch-down impressions made by current-shuffled individuals, but they are likely evidence of periodic creeping to feed via adsorption of mat nutrients^9^,^10^,^11^,^12^,^13^. This last interpretation is based on examples that show an external mould of a body fossil at the end of a serial set of faint, similar-sized casts, aligned such that the presumed anterior end of the organism is most distant from the chain of body imprints. There is also evidence of grazing and locomotory traces associated with body fossils of *Kimberella*^14^,^15^, and even signs of possible tactophobic behaviour in *Dickinsonia*^11^. Here we report new information on the problematic Ediacaran organism *Parvancorina minchami* from two bed assemblages of the Rawnsley Quartzite in South Australia, suggesting that this taxon was capable of performing rheotaxis—oriented movement or positioning in response to a water current—during at least part of its life cycle.

## Results

### Bed assemblages

**Figure 1:**
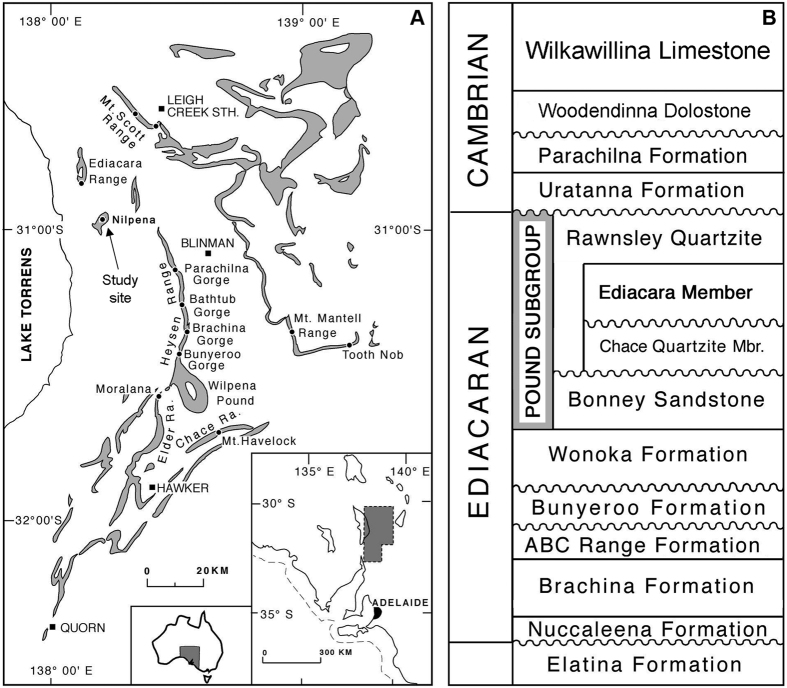
Ediacaran geology and stratigraphy of the Flinders Ranges, South Australia. (**A**) Distribution of the Pound Subgroup in the Flinders Ranges, South Australia, and location of the study site at Nilpena. Map created using Adobe Photoshop CS v.8.0 (www.adobe.com/photoshop) based on original geological mapping by James G. Gehling using aerial photographs and topographic maps. (**B**) The Ediacara Member, Rawnsley Quartzite and Pound Subgroup in the context of Ediacaran and Cambrian stratigraphy in the Flinders Ranges.

Specimens of *Parvancorina* were examined on two separate beds, Parv Bed and MM3, of the Ediacara Member of the Rawnsley Quartzite at Nilpena, Flinders Ranges, South Australia^16^ (Fig. 1). Parv Bed is a part of the ‘Planar-Laminated and Rip-Up Sandstone Facies’ that represents sub-wave base upper canyon fill deposited as unidirectional sheet-flow sands, whereas MM3 exemplifies the ‘Oscillation-Rippled Sandstone Facies’, which is interpreted to have been deposited between fair-weather and storm wave base^17^. These beds have been excavated, inverted and reassembled to study the fossil assemblages on the bed soles. On both beds, specimens of *Parvancorina* are common to abundant (relative to other beds at Nilpena^16^), preserved as negative hyporelief impressions, and are associated with a variety of other body fossils, textured organic surfaces (TOS), and sedimentary structures^1^,^18^.

**Figure 2:**
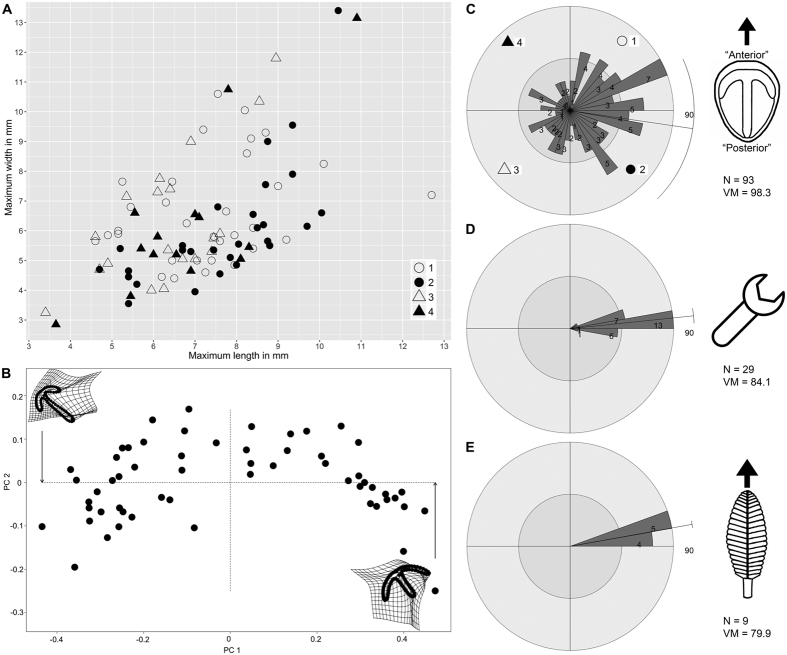
Bed assemblage data for Parv Bed. (**A**) Bivariate scatterplot of *in situ* specimens (N = 93) of *Parvancorina minchami*; different symbols correspond to numbered quadrants in (**C**). (**B**) Principal Components Analysis plot of *P. minchami* (N = 57). (**C**) Orientations of *in situ* specimens of *P. minchami*, with reconstruction showing direction of measurement. (**D**) Orientations of tool marks. (**E**) Orientations of felled frond stalks. Rose plots divided into 10° bins, showing vector mean (VM) direction; bin counts are not the same scale across plots.

**Figure 3:**
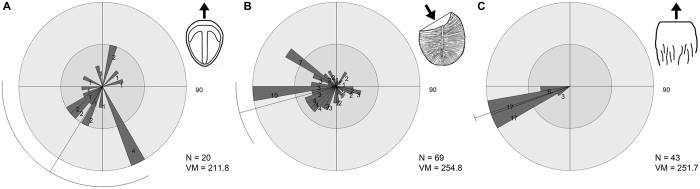
Bed assemblage data for MM3. (**A**) Orientations of *in situ* specimens of *Parvancorina minchami*. (**B**) Orientations of overfolded *Dickinsonia*, with direction measured perpendicular to fold line. (**C**) Orientations of “mop” structures. Rose plots divided into 10° bins, showing vector mean (VM) direction; bin counts are not the same scale across plots.

Parv Bed is a *c.* 7-m^2^, massive, 12–16 cm-thick, fine-medium grained sandstone bed. The planar bed sole contains a high density of well-preserved *Parvancorina minchami* (N = 93 *in situ*; *c.* 13 individuals/m^2^) representing a single cohort (Fig. 2A,B; Supplementary Figs S1–S5), showing similar ontogenetic and taphomorphic plasticity observed in the White Sea populations of *Parvancorina*^19^. Other well-preserved body fossils include *Albumares* sp., *Dickinsonia costata* and *D. lissa*, as well as *Eoandromeda octobrachiata* (see ref. 20, fig. 2E,F,I,K). Poorly or partially preserved body fossils include *Funisia dorothea*, 20–30 cm-long spicular impressions of *Coronacollina*, and felled frond stalks that show a preferred orientation (Fig. 2E). The bed sole also displays a variety of well-preserved TOS^18^. Tool mark casts also occur and indicate a strong unidirectional orientation (Fig. 2D), and are overprinted by a fine TOS indicating that the tool marks were made prior to colonization (cf. ref. 18, fig. 3A). Considering that tool marks are confined to only two sedimentary facies in the Ediacara Member, their coexistence with the fossil assemblage on Parv Bed suggests a different sequence of taphonomic events compared to the shallower wave-base facies^16^,^21^.

MM3 is a *c.* 22-m^2^, wave-rippled sandstone bed up to 6 cm in thickness. The bed sole exhibits moulds of interference ripples and a rich assemblage of body fossils and TOS, dominated by *Dickinsonia* and *Aspidella*^1^. *Parvancorina* is one of the more common constituents of the assemblage (N = 20; Fig. 3A). The bed sole also exhibits “mop”-like drag marks of uprooted frond holdfasts that show a preferred orientation^22^ (Fig. 3C). Abundant *Dickinsonia* specimens (N = 69) show varying degrees of marginal overfolding (Fig. 3B), the direction of which is remarkably consistent with the “mop” structures (see also ref. 23, fig. 2b–c). It has been clearly demonstrated that *Dickinsonia* was a thin, flexible organism that intermittently stuck to the microbial mat^11^,^24^,^25^. A preferred orientation of overfolding in *Dickinsonia* specimens on MM3 suggests that they were susceptible to currents (including those responsible for the burial event), despite having a flat body that would have loosely adhered to the substrate^23^.

Parv Bed, unlike MM3, lacks ripples. Parv Bed was therefore likely deposited below storm wave base as a proximal turbidite (sheet-flow) sand within a canyon, whereas MM3 represents event sand deposition between fair-weather and storm wave base^16^. Also, since the TOS on Parv Bed has overprinted some tool marks, as well as patches of the remaining bed sole, the tool marks record an initial scouring event that predated the colonization of the substrate by macro-organisms and TOS. Therefore, the orientations of the older tool marks and the felled frond stalks that extend into the event sand (Fig. 2D,E) record two separate events on Parv Bed involving persistent unidirectional currents that affected the substrate and its inhabitants: one prior to colonization and another at the time of burial by the event sand, respectively.

### Specimen orientations

The rose plots of *Parvancorina* specimens on Parv Bed and MM3 show a degree of scatter (Figs 2C and 3A), but statistical tests support the hypothesis that there is a preferred orientation on each bed (Supplementary Tables S1 and S2). Notably, these preferred orientations of *Parvancorina* are comparable to those of felled fronds and tool marks on Parv Bed (Fig. 2C–E), and the “mop” structures and overfolded *Dickinsonia* on MM3 (Fig. 3); circular ANOVA tests support this observation (Supplementary Tables S3 and S4). The unidirectional bias of many *Parvancorina* individuals on both beds, in association with the orientation data of various current proxies, indicates that *Parvancorina* was affected by currents either in life or just prior to burial.

## Discussion

### Taphonomy and autecology of *Parvancorina*

The life habits of *Parvancorina* have rarely been discussed in the literature. Arguments for euarthropod affinities (see refs 26 and 27 for overviews) have inferred motility, but there has been no convincing evidence for this to date^28^. Glaessner^29^ was the first to have explicitly suggested nectobenthic swimming or crawling for *Parvancorina*, based on his interpretation of fine ridges attached to the anchor-shaped structure as representing locomotory appendages (discussed further below).

There are two likely explanations for the preferred orientations of *Parvancorina* on Parv Bed and MM3, involving observations related to taphonomy and those concerning autecology. The first explanation is that individuals of *Parvancorina* were passively oriented by (and in the same direction as) the sediment-laden flow involved in their burial. The majority of facies that preserve Ediacara fossils at Nilpena consist of event beds where storm sands swept down slope from a shoreface and smothered living mat-grounds and their benthic inhabitants^16^. During such events, erect organisms such as fronds were either uprooted and dragged from the substrate (creating “mop” structures) or flattened in a preferred direction^22^. The evidence for burial-related orientations is clear on both beds considered here. On Parv Bed, frond stalks anchored by holdfasts show a strong unidirectional orientation (Fig. 2E), which closely corresponds to the mean orientation of *Parvancorina* specimens (Fig. 2C). On MM3, many *Dickinsonia* specimens have been preserved with an overfolded edge facing the current^23^ (Fig. 3B), as corroborated by “mop” orientations^22^ (Fig. 3C). Although *Parvancorina* specimens are more sparse on MM3 (N = 20; compared to Parv Bed), their mean orientation is comparable to the current proxies (Fig. 3A). However, on both beds, the notable difference between the orientations of *Parvancorina* and those of other biological or sedimentary structures is that the *Parvancorina* individuals show a higher degree of scatter. This suggests that any preferred orientation of the *Parvancorina* population is not likely the result of passive current alignment during the burial event for each bed (discussed further below).

An ecological explanation for the *Parvancorina* orientations is that individuals exhibited a rheotactic response when exposed to gentle bottom currents, either at an early stage of, or perhaps throughout their life cycle. The bedforms on Parv Bed and MM3 indicate that bottom currents may have reached flow velocities of up to 0.6 m/s during the scouring or burial events^30^, but the mean current flow velocity during calmer conditions at the time of colonization was likely to be much slower in these deep water settings below fair-weather wave base. Based on current proxies, the preferred orientation of *Parvancorina* individuals on Parv Bed and MM3 has the commonly interpreted “posterior” end of the body directed upstream (Figs 2C and 3A), assuming the ambient current direction was the same as those associated with the initial scouring or burial events on each bed. In the case of Parv Bed, the established diversity of macro-organisms and TOS suggests that there was considerable substrate exposure time between the early scouring and later burial episodes, both of which involved the same current direction (Fig. 2D,E), indicating that a persistent unidirectional bottom current probably existed during the colonization period. The “posterior” end of individuals facing up-current seems at odds with performing rheotaxis for streamlining purposes, considering the curved, elevated (up to 5 mm high) “anterior” part of the anchor structure being convex into current would have created a certain amount of drag. Moreover, if *Parvancorina* was fixed to the substrate only at the “posterior” end and free to rotate around that anchor point (like a wind sock), one would expect any influence of currents to result in a substantially less scattered distribution of individual orientations, like those of current-oriented fronds^31^,^32^ (e.g., Fig. 2E). However, *Parvancorina* shows no evidence of an attachment structure, such as a holdfast. In fact, overfolding of the “anterior” or “posterior” end has been observed in some specimens from other South Australian localities (e.g., ref. 33, fig. 18; ref. 29, pl. 3, fig. 14), indicating that the body was flexible and free-lying rather than fixed to the substrate. But there are no signs of major deformation (e.g., overfolding) of *Parvancorina* individuals on either Parv Bed or MM3, which might be expected if the currents were strong enough to shift individuals, especially during a burial event. Counteraction of drag and the ability to remain in contact with the substrate when subjected to bottom currents was probably achieved by the flat ventral surface of *Parvancorina* adpressed (i.e., stuck) to the microbial mat.

Based on the evidence presented above, it seems plausible that *Parvancorina* was a free-lying, epibenthic organism that could align itself to currents. If this is the case, rheotaxis in *Parvancorina* may relate to a number of possible functions, such as suspension feeding, respiration, disposal of metabolic waste, dispersal of gametes, or even chemosensory detection^34^,^35^,^36^,^37^,^38^, but hydrodynamic stability (i.e., station holding or streamlining to reduce energy costs) cannot be completely ruled out. Suspension feeding is one of the primary strategies for performing rheotaxis in modern sessile and motile epibenthic invertebrates^34^,^37^. However, such organisms are often attached to the substrate (permanently or temporarily) and typically possess conspicuous “filtering” structures, neither of which is obvious in *Parvancorina*. Unfortunately, there is not enough evidence to suggest that one or more of these functions best explains rheotaxis in *Parvancorina*.

The case for rheotaxis also raises the question of whether *Parvancorina* was a motile organism. There is no convincing morphological or trace fossil evidence to indicate that *Parvancorina* was either fully or facultatively motile. However, some modern invertebrates that perform rheotaxis are immobile for much of their lives, but are motile as juveniles. For example, bivalves that live in tidal settings can shift position as juveniles before becoming permanently fixed to the substrate, with adults usually retaining the orientation best suited for various functions^34^. It is also important to note that these rheotaxial bivalves show similarly scattered, but overall preferred orientations (see ref. 34, figs 7–9). It is possible that *Parvancorina* had a similar strategy, but it is also conceivable that individuals may have intermittently altered their position throughout life due to changing conditions. The best explanation for the diffuse orientations in *Parvancorina* is that they are living on a complex substrate that has a variable microtopography, with the Ediacaran seafloor covered by microbial mat irregularities, ripples (e.g., MM3), and a tiered epibenthic community, all of which are influencing fluid dynamics at (and above) the sediment-water interface^39^,^40^. Thus, individuals of low relief are likely to respond to localized turbulent flow that produces corresponding variations in orientation in space and time, rather than aligning themselves directly into the prevailing current; this hypothesis also explains the distribution of overfolded *Dickinsonia* on MM3 (Fig. 3B). The variable substrate conditions are also reflected by the disconnect between orientation and size of *Parvancorina* specimens on Parv Bed (Fig. 2A,C), and their random spatial distribution (Supplementary Fig. S5).

*Parvancorina* occurs in most facies at Nilpena, with the exception of the ‘Channelized Sandstone’ and ‘Sand-Breccia Facies’, and sandstones interpreted to have been deposited in a shoreface setting^16^. Relative abundance data indicate that *Parvancorina* is more common in the sheet-flow sands than the wave-base sands^1^, suggesting that individuals preferred lower energy, deeper water settings. The densest population at Nilpena occurs on Parv Bed (*c.* 13 individuals/m^2^) within the ‘Planar-Laminated and Rip-Up Sandstone Facies’. The higher density of individuals on the planar bed sole of Parv Bed—compared with any other bed (e.g., MM3, which preserves interference ripples sculpted by wave oscillation, with *c.* 1 individual/m^2^)—reflects calmer conditions (including an absence of wave agitation), which were better suited to the life mode of *Parvancorina*. This suggestion is further supported by a very dense bed assemblage of *Parvancorina (c.* 77 individuals/m^2^ on NECP Bed-1) from the Ediacara Conservation Park, which was deposited in the ‘Flat-Laminated to Linguoid-Rippled Sandstone Facies’^17^ (formerly the ‘Delta-Front Sand Facies’^16^), and with individuals (mostly juveniles, <5 mm long) also showing a preferred orientation to current^41^.

### Heads or tails? Morphologic and phylogenetic considerations

**Figure 4:**
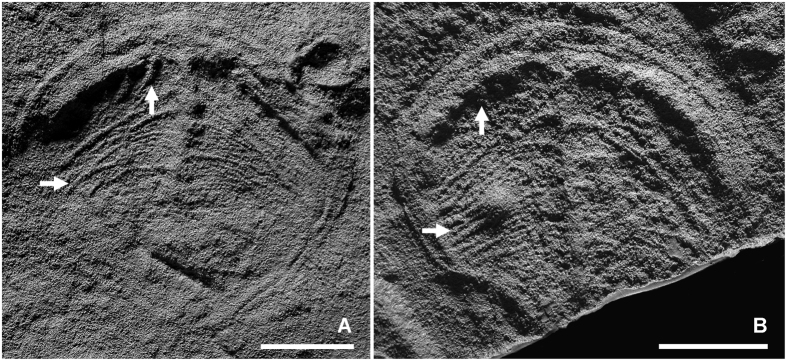
Latex moulds of large specimens of *Parvancorina minchami* from Ediacara North showing details of the longitudinal (vertical arrows) and transverse ridges (horizontal arrows) projecting from the anchor structure. (**A**) SAM P49280; (**B**) SAM P49278. Scale bars = 10 mm.

There have been a plethora of claims suggesting euarthropod affinities for *Parvancorina* (see ref. 26, table I), which have come with assumptions regarding its anterior-posterior (A-P) orientation. Some arguments have focused on the morphological similarity of the dorsal anchor-shaped structure on an undivided bilateral body with a marginal rim in Cambrian arthropods such as *Skania* and *Primicaris*^26^,^42^ (but see ref. 27 for a counterargument). Less convincing homology statements have been made when comparing *Parvancorina* with the single-tagma protaspides of trilobites, specifically the broad similarity of the anchor structure in the former with the shape of the connected glabella and eye ridges in the latter. Some authors^29^,^33^,^43^,^44^ have also interpreted the fine ridges that project from the anchor structure on some specimens of *Parvancorina* as appendages (Fig. 4; ref. 29, pl. 1, figs 1–3, pl. 2, figs 4–6, pl. 3, figs 15 and 16). However, this interpretation has been criticized, with Hou *et al*.^45^ and Legg^27^ correctly pointing out that the series of ridges projecting from the interior-facing edge of the curved, transverse portion of the anchor structure (i.e., Glaessner’s^29^,^33^ “anterior appendages”; referred to here as longitudinal ridges [Fig. 4]), is incompatible with an arthropod body plan; this also negates the suggested homology of this structure with the eye ridges of trilobite protaspides. Moreover, the ridges that project from the axial portion of the anchor structure (i.e., Glaessner’s^29^,^33^ “posterior appendages”; referred to here as transverse ridges [Fig. 4]), often interdigitate or overlap (e.g., ref. 29, pl. 2, fig. 6), which again is inconsistent with arthropod appendage morphology, particularly the disposition of appendages in *Skania* (ref. 26, figs 5 and 6; ref. 27, fig. 1B) and *Primicaris* (ref. 42, text-figs 1, 2, 5 and 6). Whilst there are some superficial similarities to acercostracan euarthropods^27^, based on available evidence, there are no robust characters that confirm placement of *Parvancorina* within the Euarthropoda.

New taphonomic and autecological information also brings into question the A-P orientation of *Parvancorina*. As part of his Vendobionta model, Seilacher^24^ radically deviated from previous interpretations by turning *Parvancorina* on its “head” and reconstructed it as an erect, frond-like organism having a unipolar growth pattern, with the supposed “anterior” end of the anchor structure representing the holdfast. Whilst the longitudinal ridges appear to show a regular pattern (discussed below), the often chaotic appearance of the transverse ridges does not support unipolar growth characterized by serial addition of “quilts” at one end. Also, the orientation data of *Parvancorina* relative to the presumed current direction (especially on Parv Bed) defy typical biostratinomic patterns for current-aligned fronds (discussed above). It has also been noted that *Parvancorina* looks superficially similar to the anterior anchor-like structure seen in some specimens of *Kimberella*^46^, with the insinuation that *Parvancorina* is not an entire organism, but rather a body part. This interpretation is easily refuted, as the anchor-like structure in *Kimberella* merges with the main axial part of the body and is not surrounded by a marginal rim^14^. Unfortunately, it is not possible to determine the A-P orientation of *Parvancorina* based on rheotactic behaviour alone, as it is common for organisms to perform positive and negative rheotaxis, with the anterior of the organism pointing upstream or downstream, respectively.

While the orientation of the A-P axis in *Parvancorina* remains an open question, its network of longitudinal and transverse ridges (Fig. 4) might have played a functional role related to its rheotactic behaviour if one or both sets of ridges are, in fact, external features. This interpretation would have the ridges being directly exposed to the current, since the “posterior” end of the body is often pointing upstream. Notably, the orientation, regular spacing and higher relief of the longitudinal ridges (when preserved, see especially ref. 29, pl. 1, figs 2 and 3; ref. 47, fig. 3a) would seem to be ideal structures to help channel water flow. However, given that both sets of ridges are not often preserved, particularly in smaller specimens (<10 mm in length), complicates the issue of whether they are truly external, or indeed real morphological structures. This leaves two other alternatives: (1) if the network of ridges is internal, it may represent some sort of coelomic system, thus suggesting that *Parvancorina* was a triploblastic grade animal; or (2) the ridges are simply a taphonomic artefact that have resulted from wrinkling or contraction of the body. Unfortunately, based on the available evidence, the identity of these ridges remain problematic.

## Conclusions

This study further reinforces the importance of examining large Ediacaran bedding surfaces in order to decipher the taphonomic history, palaeobiology and ecology of these enigmatic organisms and the range of palaeoenvironmental conditions under which they lived^1^,^48^,^49^,^50^. In some cases, such field-based approaches may be the only way of discovering novel palaeobiological information on the Ediacara biota, including details on their locomotion, feeding and reproduction. Whilst rheotaxis in *Parvancorina* does not in itself provide unequivocal evidence of full or facultative motility, a rheotactic lifestyle does shed new light on the ecological complexity of a largely immobile Ediacaran community. Although different kinds of motility and associated ecospace modes amongst epibenthic organisms are largely a Phanerozoic phenomenon linked to the diversification of bilaterians during the Cambrian^51^, there appears to be more movement among Ediacaran taxa than previously realized.

A better understanding of the palaeoecology of an enigmatic organism can sometimes help to reveal its phylogenetic affinity, but such claims should always be supported by the presence of key (unambiguous) morphological features. The anatomical fidelity of *Parvancorina*, like many other members of Ediacara biota, has suffered from a complex taphonomic history within coarse-grained siliciclastic sediments^52^. Thus, apart from possessing a bilaterally symmetrical body, there are no unequivocal morphological characters to support placement of *Parvancorina* within the Bilateria, though this possible affinity is not completely at odds with the available trace fossil, phylogenetic and molecular clock evidence for bilaterians in the Ediacaran^53^,^54^,^55^,^56^.

## Methods

### *Parvancorina* orientations

To demonstrate whether *Parvancorina* specimens have a preferred orientation on Parv Bed and MM3, we employed the Chi-square test (using the ‘stats’ package), in addition to the Rayleigh Test of Uniformity and Kuiper’s Test of Uniformity (both run using the ‘circular’ package) in R^57^,^58^. The tests considered the following hypotheses:

For Parv Bed (Supplementary Table S1):

- Null hypothesis: The *Parvancorina* specimens have no preferred orientation.
- Alternative hypothesis: The *Parvancorina* specimens have a preferred orientation.

For MM3 (Supplementary Table S2):

- Null hypothesis: The *Parvancorina* specimens have no preferred orientation.
- Alternative hypothesis: The *Parvancorina* specimens have a preferred orientation.

### Circular ANOVA test

This test was used to determine if the mean orientations of *Parvancorina* and associated current proxies (e.g., tool marks, felled fronds, overfolded *Dickinsonia* and “mop” structures) on Parv Bed and MM3 are statistically comparable. Tests were run in an R environment using the packages ‘circular’ and ‘Directional’^57^,^58^,^59^. The functions ‘aov.circular’, ‘hcf.circaov’, ‘lr.circaov’, ‘het.circaov’ were used to test the following hypotheses:

For Parv Bed (Supplementary Table S3):

- Null hypothesis: The mean orientations of *Parvancorina*, tool marks and felled fronds are equal.
- Alternative hypothesis: The mean of the orientations of at least one of *Parvancorina*, tool marks and felled fronds is different.

For MM3 (Supplementary Table S4):

- Null hypothesis: The mean orientations of *Parvancorina*, overfolded *Dickinsonia* and “mop” structures are equal.
- Alternative hypothesis: The mean of the orientations of at least one of *Parvancorina*, overfolded *Dickinsonia* and “mop” structures is different.

### Bayesian Information Criterion (BIC) analysis

The bivariate dataset of maximum length and width measurements of *Parvancorina* specimens on Parv Bed (Fig. 2A) was analysed using the MCLUST clustering analysis in R to assess whether one or more groups exist within the sample^60^. The approach of Darroch *et al*.^61^ in conducting univariate analyses of a multivariate dataset was not followed here as their approach results in multiple testing, thus ‘equal’ and ‘unequal’ variance plots have not been produced. A single multivariate BIC plot showing five cluster models using combinations of constrained (E) and unconstrained (V) shape, volume and orientation was produced (Supplementary Fig. S2); four of these models (EEE, EEV, VEV, VVV) were included by Darroch *et al*.^61^.

### Morphometrics

A geometric morphometric analysis was conducted on a subset of *Parvancorina* specimens (N = 57) from Parv Bed in order to assess morphological variation; the remainder of the specimens from Parv Bed (N = 36) are not preserved well enough for the purposes of accurate landmarking and semi-landmarking. To conduct the semi-landmark analysis, the following steps were taken: Specimen latexes were coated with ammonium chloride, then photographed using a Canon EOS 5D digital camera with a Canon MP-E 65 mm 1–5x macro lens. Landmarking and semi-landmarking (see Supplementary Fig. S4) was conducted using the Thin-Plate Spline (tps) suite (http://life.bio.sunysb.edu/morph/index.html). A tps file was constructed using tpsUtil64 (v.1.7)^62^. The tps file was imported into tspDig2 (v.2.26)^63^, which was used to place the five landmarks and 80 semi-landmarks around the *Parvancorina* anchor structure and populate the tps file with the semi-landmark and landmark data. The tps file was imported into an R environment^58^. The ‘geomorph’ package^64^ was used to conduct the Procrustes Superposition and Principal Components Analysis (PCA) of the superimposed data, producing Fig. 2B.

## Additional Information

**How to cite this article**: Paterson, J. R. *et al*. Rheotaxis in the Ediacaran epibenthic organism *Parvancorina* from South Australia. *Sci. Rep.* **7**, 45539; doi: 10.1038/srep45539 (2017).

**Publisher's note:** Springer Nature remains neutral with regard to jurisdictional claims in published maps and institutional affiliations.

## Supplementary Material

Supplementary Information
