# Supplementary material for: Rheotaxis in the Ediacaran epibenthic organism Parvancorina from South Australia
Source: Sci Rep. 2017 Mar 30;7:45539. doi: 10.1038/srep45539 (PMC5371987; doi:10.1038/srep45539)
Supplement: Supplementary Information [file srep45539-s1.pdf]

Supplementary Information

Rheotaxis in the Ediacaran epibenthic organism *Parvancorina* from South Australia

John R. Paterson[1*], James G. Gehling[2], Mary L. Droser[3] and Russell D.C. Bicknell[1]

[1]Palaeoscience Research Centre, School of Environmental and Rural Science, University of New England, Armidale, NSW 2351, Australia

[2]South Australian Museum, North Terrace, Adelaide, SA 5000, Australia

[3]Department of Earth Sciences, University of California, Riverside, California 92521, USA

* E-mail: jpater20@une.edu.au

CONTENTS:

Supplementary Tables S1–S4

Supplementary Figures S1–S5

Supplementary References

SUPPLEMENTARY TABLES

**Test name**	**Test statistic**	**p-value**
**Chi-square test**	5616.2	< 2.2e-16
**Rayleigh Test of Uniformity**	0.2466	0.0035
**Kuiper's Test of Uniformity**	2.014	< 0.01

Table S1. The results of the tests considering whether *Parvancorina* specimens have a preferred orientation on ParvBed. All tests illustrate that at the 5% level there is a statistically significant preference for orientation and, as such, the null hypothesis is rejected in favour of the alternative hypothesis: *Parvancorina* specimens on ParvBed have a preferred orientation. These tests do not indicate the preferred orientation. However, the rose plot in Figure 2C shows that approximately two-thirds of the *Parvancorina* specimens on ParvBed are oriented towards the right-side quadrants, illustrating that the preferred orientation relates to the vector mean.

Test name	Test statistic	p-value
**Chi-square test**	810.77	< 2.2e-16
**Rayleigh Test of Uniformity**	0.3517	0.083
**Kuiper's Test of Uniformity**	0.025	< 0.05

Table S2. The results of the tests considering whether *Parvancorina* specimens have a preferred orientation on MM3. The Chi-square test and Kuiper's Test of Uniformity indicate at the 5% level there is a statistically significant preference for orientation and as such the null hypothesis is rejected in favour of the alternative hypothesis for these two tests: *Parvancorina* specimens have a preferred orientation. These tests do not indicate the preferred orientation. However, the rose plot in Figure 3A shows that 50% of the *Parvancorina* specimens on MM3 are orientated towards the bottom left quadrant, illustrating that the preferred orientation relates to the vector mean. The reason why the Rayleigh Test of Uniformity failed to reject the null hypothesis most likely relates to the sample size (N=20) being too small to successfully resolve a preferred orientation.

Analysis	Package and function	Test statistic	p-value
**Circular ANOVA High Concentration F-Test**	Circular, aov.circular	0.6414	0.52830
**High concentration F-Test**	Directional, hcf.circaov	0.640660	0.5286221
**Log-likelihood ratio F-Test**	Directional, lr.circaov	1.0698382	0.5857167
**Non equal concentration parameters approach F-Test**	Directional, het.circaov	5.84374254	0.05383286

Table S3. Four circular ANOVA tests of the ParvBed orientation data. These analyses illustrate that *Parvancorina* specimens, felled fronds and tool marks have a statistically similar orientation on ParvBed, with all p-values greater than 0.05. The null hypothesis is therefore not rejected and it is realised that the three groups have statistically similar mean orientations, at the 5% level.

Analysis	Package and function	Test statistic	p-value
**Circular ANOVA High Concentration F-Test**	Circular, aov.circular	2.582	0.07956
**High concentration F-Test**	Directional, hcf.circaov	2.5793	0.07973
**Log-likelihood ratio F-Test**	Directional, lr.circaov	4.9802	0.08290
**Non equal concentration parameters approach F-Test**	Directional, het.circaov	2.5547	0.2788

Table S4. Four circular ANOVA tests of the MM3 orientation data. These analyses illustrate that *Parvancorina* specimens, "mop" structures and overfolded *Dickinsonia* specimens have a statistically similar orientation on MM3, with all p-values greater than 0.05. The null hypothesis is therefore not rejected and it is realised that all groups all have statistically similar mean orientations, at the 5% level.

SUPPLEMENTARY FIGURES

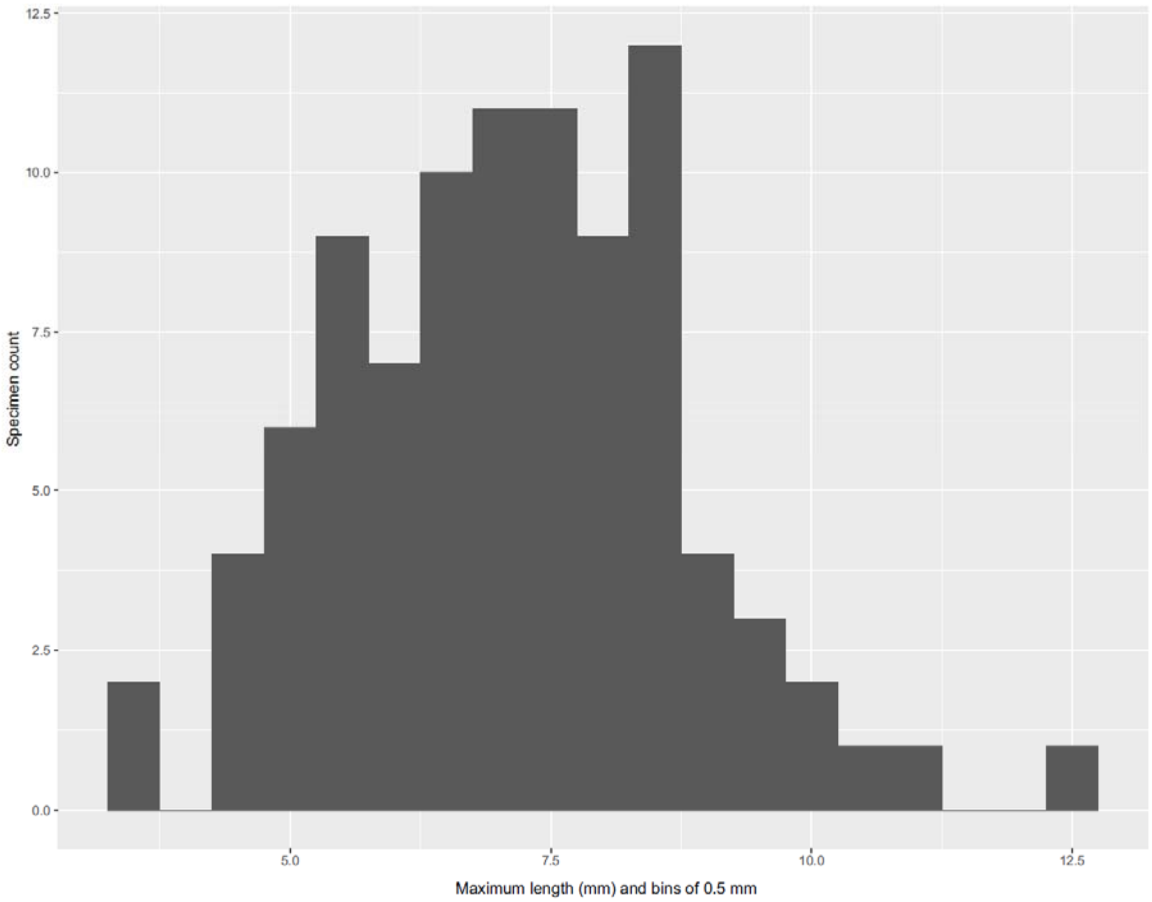

Figure S1. Histogram of the maximum length of *Parvancorina* specimens (N=93) from ParvBed. The unimodal distribution of lengths, ranging between 3.5 and 12.5 mm, illustrates one population with substantial size variation. Figure constructed in R using ‘ggplot2’ and ‘reshape2’ (Wickham, 2007, 2009).

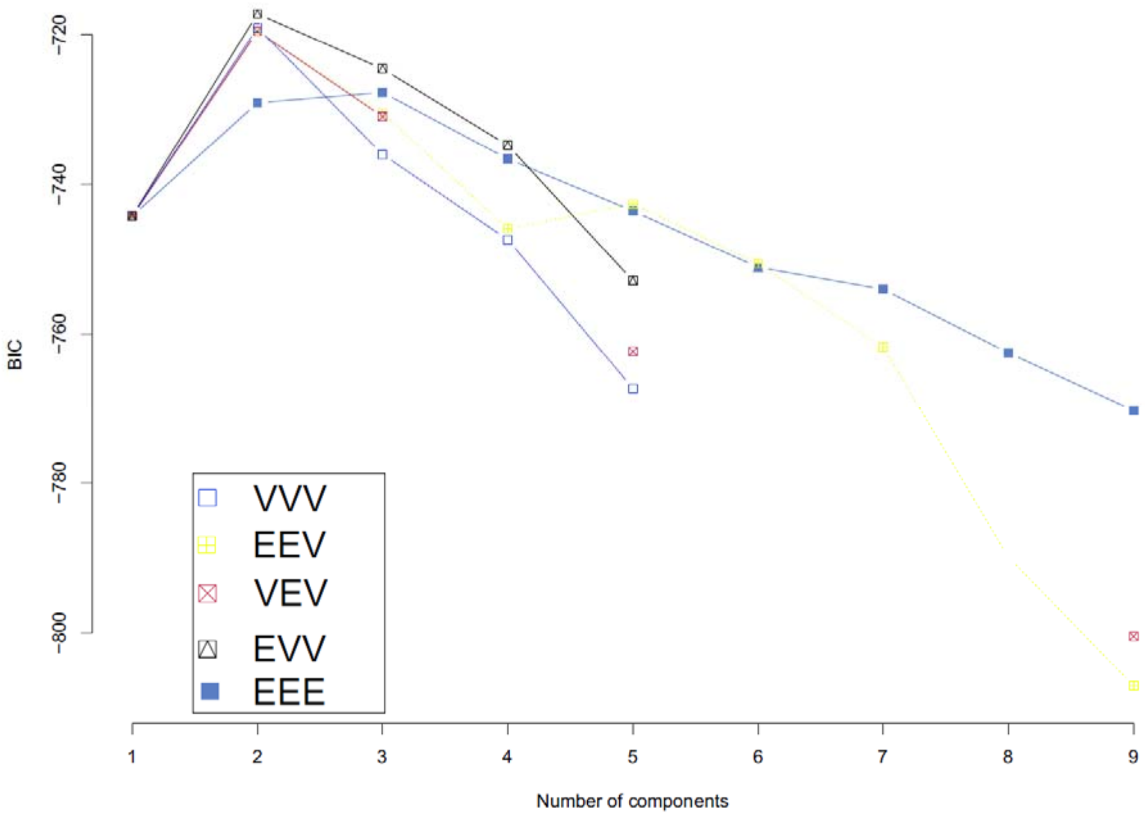

Figure S2. The BIC plot constructed using the bivariate dataset of maximum width and length measurements of *Parvancorina* specimens from ParvBed (N=93). The BIC analysis suggests that the value of ‘2’ components best describes the construct of the studied assemblage. Taken at face value, and without considering detailed morphology or taphomorphic variability, this suggests that there are possibly two populations or perhaps two species in the studied sample. However, the geometric morphometric study conducted here (Fig. 2B) would indicate that there is a morphological continuum of specimens that represent only one population of a single species. The value of ‘2’ is likely the result of an arbitrary division forced by the cluster analysis (see Fig. S3 below). Plot was constructed in R using MCLUST (Fraley et al., 2012).

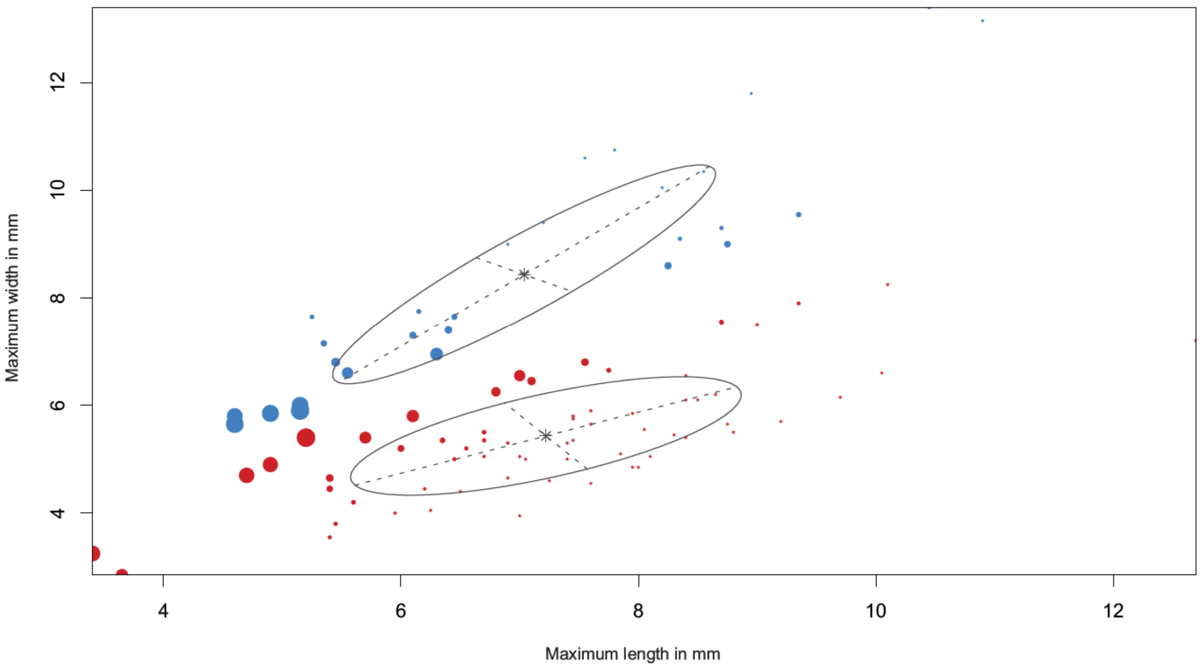

Figure S3. The uncertainty plot relating to the BIC analysis (see Fig. S2 above). This plot illustrates that the delineation between the two clusters (blue and red dots) is arbitrary, especially given the high level of uncertainty surrounding several specimens (large dots) at the supposed cluster boundary. Thus, the division between the two clusters (or components) is an artefact of the cluster analysis and does not represent a true biological signal. Plot produced in R using MCLUST (Fraley et al., 2012).

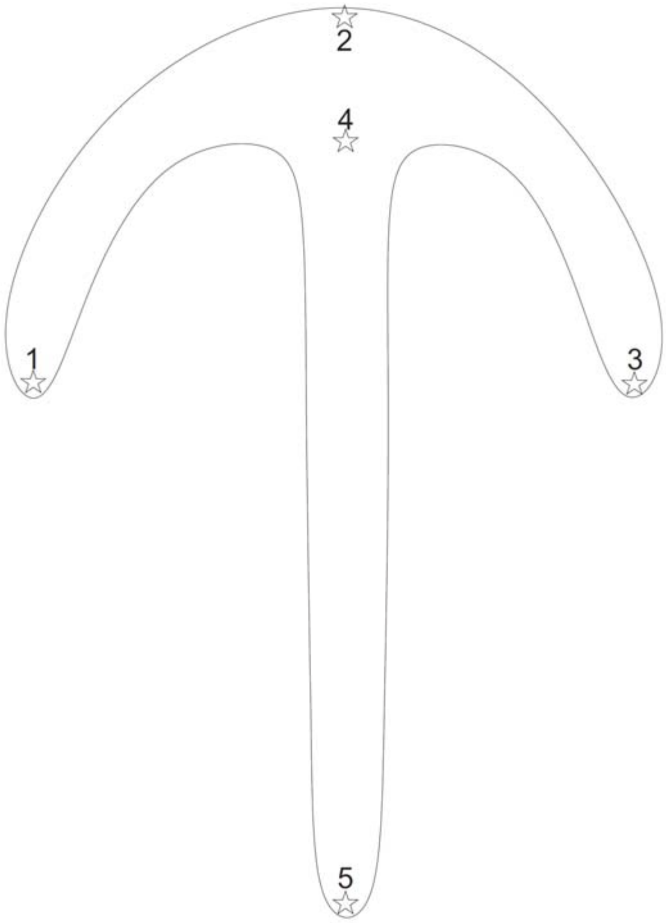

Figure S4. An outline drawing of the *Parvancorina* anchor structure showing the five landmark localities (stars) labelled in sampling order. Semi-landmarks were used to construct an outline of the anchor structure for the best preserved specimens (N=57) on ParvBed and used for the geometric morphometric analysis (Fig. 2B). The semi-landmarked outline for all specimens started at the first landmark (1) and moved clockwise around the edges of the anchor structure, and closed on the first landmark.

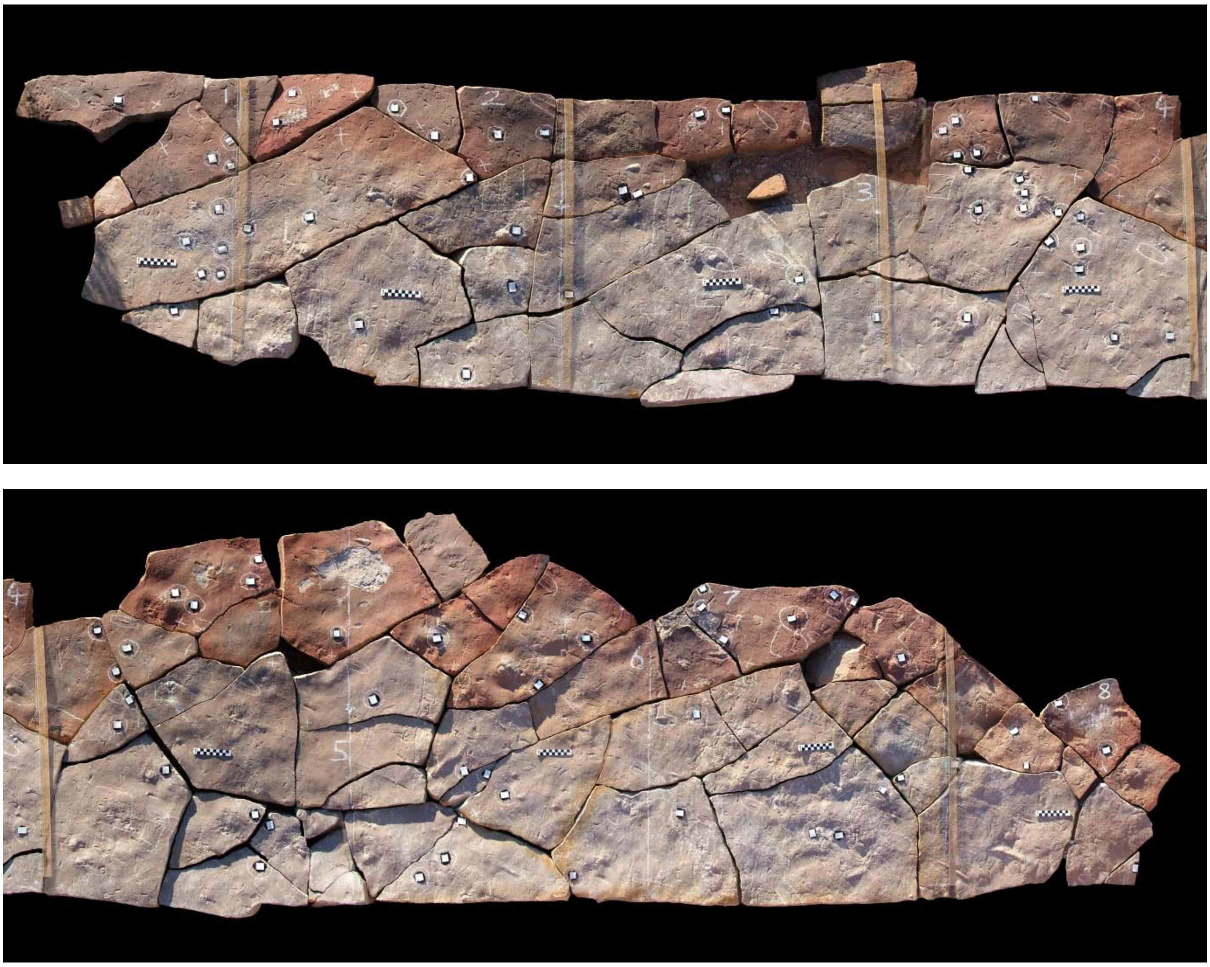

Figure S5. Stitched panorama of ParvBed (top image: western half; bottom image: eastern half) showing spatial distribution of *Parvancorina* specimens (indicated by white square labels). Scale bars in centimeters.

SUPPLEMENTARY REFERENCES

Fraley, C., Raftery, A.E., Murphy, T.B., and Scrucca, L., 2012, MCLUST, Version 4 for R: Normal Mixture Modeling for Model-Based Clustering, Classification, and Density Estimation, Technical Report No. 597, Department of Statistics, University of Washington.

Wickham, H., 2007, Reshaping Data with the reshape Package: Journal of Statistical Software, v. 21, p. 1–20, URL: http://www.jstatsoft.org/v21/i12/.

Wickham, H., 2009, ggplot2: Elegant Graphics for Data Analysis. Springer-Verlag New York.
